# Social capital and loneliness among older adults in community dwellings and nursing homes in Zhejiang Province of China

**DOI:** 10.3389/fpubh.2023.1150310

**Published:** 2023-05-19

**Authors:** Yan Chen, Yuchen Zhou, Min Li, Yanyan Hong, Hongkun Chen, Shanshan Zhu, Yiying Zhou, Shuangyu Yang, Xianlan Wu, Dahui Wang

**Affiliations:** School of Public Health, Hangzhou Normal University, Hangzhou, Zhejiang, China

**Keywords:** social capital, loneliness, older adult, nursing home, community

## Abstract

**Background:**

Loneliness is an important problem afflicting the health of older adults, and has been proven to be associated with social capital. Previous research in China rarely investigated the differences of social capital and loneliness between older adults living in community dwellings and nursing homes. This study aims to examine the status of social capital and loneliness among older adults living in community dwellings and nursing homes, and analyze the relationship between them.

**Methods:**

A total of 1,278 older adults were recruited for the study from the cities of Hangzhou, Huzhou, and Lishui in Zhejiang Province of China from July to October 2021 by using multi-stage stratified random sampling. Questionnaires were used to collect data on the participants’ sociodemographic characteristics, social capital, and loneliness. Hierarchical multiple regression was used to examine the relationship between social capital and loneliness. The interaction of social capital and institutionalization on loneliness was also explored.

**Results:**

Compared with community-dwelling older adults, institutionalized older adults had higher levels of loneliness and lower degrees of social support, social connection, trust, cohesion, and reciprocity. A further analysis of the social capital showed that low levels of social support, trust, and cohesion were related to high levels of loneliness among adults in both community dwellings and nursing homes. Social connection was negatively correlated with loneliness among older adults living in community dwellings. Institutionalization itself demonstrated a strong effect on loneliness.

**Conclusion:**

Health-related policies should help older adults gain more social support, trust and cohesion to alleviate their loneliness. This is particularly crucial for older adults living in nursing homes, as they have higher levels of loneliness and lower levels of social capital than noninstitutionalized older adults.

## Introduction

As a negative subjective emotional state, loneliness is an important and prevalent problem afflicting the health of older adults (1), and is prevalent among this population across countries. A US study found that 43% of older adults experienced loneliness (2). A research found that approximately 19.6–34.0% of older adults in Europe experienced loneliness (3). Data from the Chinese Longitudinal Healthy Longevity Survey (CLHLS) showed that about 53.5% of older adults suffered from a feeling of loneliness (4). Because loneliness can impair the physical and mental health of older adults to reduce their quality of life (5, 6). Advanced age (3), level of education (7), marital status (8), monthly income (9), and living environment (10) are the influencing factors of loneliness. Prevention strategies should be developed to recognize the risk factors of loneliness and deal with its adverse outcomes.

Social capital, as a social resource for mental health promotion (11, 12), can actually alleviate the levels of loneliness in older adults (10, 13). Social capital was first proposed by Bourdieu (14), who describes it as an ensemble of social network resources. According to Putnam (15) and Coleman (16), one of the most widely used definitions of described social capital as consisting of the features of social organizations, including social networks, norms, and trust, which can improve social efficiency by promoting coordinated actions. Typically, social capital is regarded as the quantity (frequency) and quality (perceived connections) of social networks (10, 17), including both structural social capital and cognitive social capital (18). Social participation, social support, and social connection can be categorized as dimensional components of structural social capital, while trust, cohesion, and reciprocity are dimensional components of cognitive social capital. The measurement of social capital has evolved from a single dimension (7, 10) in the past to a current tendency to measure multiple dimensions (17, 19, 20). In this study, we used a multidimensional social capital scale to examine the effects of structural social capital and cognitive social capital on loneliness in older adults, respectively.

As age and physical activity decline, social capital, as practical help and support for older people in their daily lives, has received more attention from researchers (17). Social capital theory can help researchers understand the impact of differences in place of residence on mental health and well-being (21). Most empirical studies concluded that social capital and loneliness are context dependent, such as geographical location or residential settings (10, 22). A study from the UK investigated the effects of housing with care on loneliness and found that those who lived in housing with care experienced lower levels of loneliness than would be expected if they lived in the general community (23). Another study in China compared social capital indicator factors on loneliness among widowed older adults between rural and urban areas. Compared to urban areas, widowed older adults in rural areas have lower social capital and higher levels of loneliness. It was found that social connection, trust, and cooperation were strongly associated with loneliness among widowed older adults in rural areas, but not among those in urban areas (24).

Similarly, institutions and non-institutions presented differences, with higher loneliness and lower social capital commonly found among institutionalized older adults (7, 10, 25, 26). Existing studies have analyzed the correlation between social connection and loneliness in older adults, presenting inconsistent results in institutionalized and noninstitutionalized living settings. A study from Norway reported that low levels of social connection (frequent contact with family members, friends, or neighbors) was associated with higher levels of loneliness among noninstitutionalized residents, but this association was not found among institutionalized residents (7). Differently, a study from Spain reported that social connection, like gathering with family members, friends, or neighbors, was associated with loneliness among residents in institutional settings, but not in noninstitutionalized settings (11, 12). However, a Finland study revealed that social connection was not linked with loneliness among older adults in both institutionalized and noninstitutionalized living settings (10). Maybe there are many reasons to explain this inconsistency, including different cultural background (7), and different scales used in different countries (20). Earlier research on the relationship between social capital and loneliness in institutionalized and noninstitutionalized older adults focused on western developed countries. Given the Eastern cultural background, it should be explored whether there are variations between China and western nations in the associations between them.

China has the largest aging population in the world (27), and aged care modes for older adults mainly include noninstitutionalized and institutionalized care. According to data from the Ministry of Civil Affairs of China (28), about 97% of Chinese older adults lived in communities for aging, and 3% lived in institutions for aging. According to the Chinese cultural background, the Chinese older adults prefer living in their homes, which enable them to easily connect with their family members, friends, and neighbors (29). Moving into a nursing home represents a departure from their familiar neighborhood and a disruption of the close social networks they had before (30, 31), which probably led to decreased social capital and an increased risk for loneliness. However, previous studies (7, 32, 33) in China mainly focused on the relationship between social capital and loneliness in noninstitutionalized older adults, with few concentrations on institutionalized older adults or comparative studies between both institutionalized and noninstitutionalized settings.

Considering that it is theoretically and practically important to explore the differences in social capital between both care modes, and the effect of social capital on loneliness among older adults, which is meaningful for improving nursing services, and developing health-related policies. The purpose of this study was to investigate the effects of institutionalization on loneliness among older adults and analyze the relationship between six components of social capital (social participation, social support, social connection, trust, cohesion, and reciprocity) and loneliness among institutionalized and noninstitutionalized older adults. Therefore, we proposed the following hypotheses: (i) Social capital is negatively associated with loneliness among older adults, and this applies to each dimension of social capital and for both institutionalized and noninstitutionalized older adults; (ii) Older adults living in nursing homes have higher levels of loneliness than those living in community dwellings; (iii) Social capital has a stronger effect on loneliness for older adults living in nursing homes than those living in community dwellings.

## Materials and methods

### Study design and data collection

We conducted a cross-sectional survey from July to October of 2021 in Zhejiang Province, China. According to China’s seventh population census in 2021, 23.43% of the total population in Zhejiang Province is 60 years old or above (34), which is higher than the average level of China (18.70%). A multi-stage stratified cluster sampling method was applied. First, we selected three cities based on economic level—Hangzhou (high economic level), Huzhou (middle economic level), and Lishui (low economic level); Second, three districts were randomly selected from each city based on a high, middle, or low level of urbanization; Third, one community dwelling and one nursing home were randomly selected from each district, resulting in a total of nine nursing homes and nine communities chosen for the survey.

A total of 1,440 respondents (about 80 older adults from each community and each nursing home) were enrolled in the survey and accepted a face-to-face interview by convenience sampling with the help of leaders in charge of the community and institutions. A team of eight college students from the School of Public Health, Hangzhou Normal University conducted face-to-face interviews after receiving training. The criteria for including participants were as follows: (1) age ≥ 60 years, (2) lucidity, and (3) adequate capabilities of comprehension and communication with the investigators. When the surveys were finished, gifts (approximately 1.5 US dollars) were rewarded for the respondents. Finally, a total of 1,278 questionnaires (670 from nine nursing homes and 608 from nine community dwellings) were acquired, of which 162 were incomplete due to temporary health conditions (45), unwillingness to invest time in the interview (78), and other unspecified reasons (39). The valid acceptance rate of the questionnaire was 88.75%.

### Measures

#### Measurement of loneliness

Loneliness was measured by the short-form UCLA Loneliness Scale (ULS-8) as adapted by Hays et al. (35). ULS-8 contains eight items, each of which is scored on a four-point Likert scale (1 = never, 2 = seldom, 3 = usually, 4 = always), and two items were reverse-coded prior to analyses. The total score ranged from eight to 32 points, and a higher score indicated greater feeling of loneliness experienced by the relevant respondent. The value of Cronbach’α on ULS-8 among older adults was 0.831 (36), and its internal reliability in our study was 0.913. Details about the measurement of loneliness please refer to Supplementary Table S1.

#### Measurement of social capital

Based on the World Bank’s Social Capital Assessment Tool and the related literature (37, 38), we included cognitive social capital (trust, cohesion, and reciprocity) and structural social capital (social participation, social support, and social connection) in this study. We used an adapted Chinese version of this assessment tool containing 22 items (Supplementary Table S2). The items were scored using a five-point Likert scale (1 = never, 2 = seldom, 3 = usually, 4 = often, 5 = more often). The value of Cronbach’s α for the scale was 0.919 in the previous study (39), and was 0.879 in our study.

The score for each dimension was calculated as the sum of the scores of each item along that dimension. Binary variables (high and low levels) were generated, and categorized into two groups for each dimension according to their relative median values for analysis (40, 41). They included social participation [high (≥6) and low (<6)], social support [high (≥13) and low (<13)], social connection [high (≥12) and low (<12)], trust [high (≥13) and low (<13)], cohesion [high (≥20), and low (<20)], and reciprocity [high (≥11) and low (<11)].

#### Covariates

Sociodemographic and health-related variables, including age, gender, level of education, marital status, monthly income, whether or not the subject had chronic diseases, and number of children, were collected through standardized questionnaires. The level of education was defined as 1–6 years if the subject had attended primary school, 7–9 years for secondary school, and 10–12 years for high school. Marital status included married and others (single, widowed, and divorced). Chronic diseases were measured according to whether the subject had ever been diagnosed with diseases of this kind by a healthcare professional or had a record of taking medication for them. Monthly income was the ratio of total monthly household income to the population of the household. The variables were coded as follows: age (70–79 and 80+ each coded 1 vs. 60–79 = 0), sex (female = 1 vs. male = 0), education (middle and high or above each coded 1 vs. primary or below = 0), marital status (others = 1 vs. married = 0), monthly income (RMB) (3000–4,999 and 5,000–9,999 and 10,000+ each coded 1 vs. 0–2,999 = 0), number of children (1–2 and 3+ children each coded 1 vs. none), number of chronic conditions (1 and 2+ each coded 1 vs. none), care mode (living in nursing home = 1 vs. living in the community = 0).

### Statistical analysis

The categorical variables were expressed in numbers (%) and the continuous variables were expressed as mean ± standard deviation. The differences between older adults living in nursing homes and those living in community dwellings were compared by using the chi-square test for categorical variables and *T*-test for continuous variables. Hierarchical multiple regression was applied to analyze the influence of institutionalization, social capital, and the interaction of social capital and institutionalization on loneliness among older adults. Variables such as their age, level of education, marital status, number of chronic diseases, and monthly income were adjusted in the regression model. All analyses were processed by using SPSS 23.0 statistical software (IBM Corp., Armonk, NY, United States). *p* < 0.05 was taken as representative of statistical significance.

## Results

### Results of descriptive analysis

Descriptive information of the studied population is presented according to sociodemographic, diseases, social capital, and loneliness (Table 1). In terms of sociodemographic, age, education, monthly income, and marital status were statistically different between residents in community dwellings and nursing homes (*p* < 0.001). Regarding diseases, seniors living in nursing homes had significantly higher rates of chronic diseases than those living in community dwellings (85.8% vs. 64.8%, *p* < 0.001).

**Table 1:** Characteristics of the study participants.

Variable	Total*n* = 1,278	Older adults in community dwellings*n* = 608	Older adults in nursing homes*n* = 670	χ^2^ or *t*	*p*
Age				136.025	<0.001
60–69	491 (38.4)	307 (50.5)	184 (27.5)		
70–79	445 (34.8)	226 (37.2)	219 (32.7)		
80-	342 (26.8)	75 (12.3)	267 (39.9)		
Sex				0.378	0.539
Male	554 (43.3)	269 (44.2)	285 (42.5)		
Female	724 (56.7)	339 (55.8)	385 (57.5)		
Education				19.688	<0.001
Primary or below	687 (53.8)	292 (48.0)	395 (59.0)		
Middle	308 (24.1)	152 (25.0)	156 (23.3)		
High or above	283 (22.1)	164 (27.0)	119 (17.7)		
Marital status				123.843	<0.001
Married	762 (59.6)	460 (75.7)	302 (45.1)		
Others	516 (40.4)	148 (24.3)	368 (54.9)		
Monthly income (RMB)				167.588	<0.001
0–2,999	376 (29.4)	135 (22.2)	241 (36.0)		
3,000–4,999	468 (36.6)	162 (26.6)	306 (45.7)		
5,000–9,999	323 (25.3)	219 (36.0)	104 (15.5)		
10,000-	111 (8.7)	92 (15.1)	19 (2.8)		
Number of children				0.350	0.983
0	23 (1.8)	11 (1.8)	12 (1.8)		
1–2	1,057 (82.7)	499 (82.1)	558 (83.3)		
3-	198 (15.5)	98 (16.1)	100 (14.9)		
Having chronic diseases				76.811	<0.001
No	309 (24.2)	214 (35.2)	95 (14.2)		
Yes	969 (75.8)	394 (64.8)	575 (85.8)		
Structural social capital					
Social participation				0.000	0.987
Low	645 (50.5)	307 (50.5)	338 (50.4)		
High	633 (49.5)	301 (49.5)	332 (49.6)		
Social support				131.215	<0.001
Low	629 (49.2)	197 (32.4)	432 (64.5)		
High	649 (50.8)	411 (67.6)	238 (35.5)		
Social connection				31.440	<0.001
Low	1,167 (91.3)	527 (86.68)	640 (95.52)		
High	111 (8.7)	81 (13.32)	30 (4.48)		
Cognitive social capital					
Trust				115.351	<0.001
Low	854 (66.8)	316 (52.0)	538 (80.3)		
High	424 (33.2)	292 (48.0)	132 (19.7)		
Cohesion				142.136	<0.001
Low	750 (58.7)	252 (41.4)	498 (74.3)		
High	528 (41.3)	356 (58.6)	172 (25.7)		
Reciprocity				111.170	<0.001
Low	548 (43.0)	168 (27.6)	381 (56.9)		
High	729 (57.0)	440 (72.4)	289 (43.1)		
Loneliness	15.67 ± 5.69	13.78 ± 5.38	17.40 ± 5.40	−11.982	<0.001

Moreover, the ratio of residents with low social capital in nursing homes was greater than those in community dwellings: social support (64.5% vs. 32.4%), and social connection (95.52% vs. 86.68%), trust (80.3% vs. 52%), cohesion (74.3% vs. 41.4%), reciprocity (56.9% vs. 27.6%). There were no statistical differences in social participation (50.5% vs. 50.4%, *p* > 0.05) between two groups. Finally, a significant difference in loneliness was found between both groups: older adults living in nursing homes experienced loneliness more frequently than those living in community dwellings (17.40 and 13.78, respectively, *p* < 0.001).

### The influence of social capital and institutionalization on loneliness

Hierarchical multiple regression was used to analyze the effects of social capital and institutionalization on loneliness among older adults in model 1, model 2, model 3, and model 4 (Table 2). In model 1 (unadjusted), loneliness among the participants was significantly associated with social support (*β* = −1.120, *p* < 0.001), social connection (*β* = −2.716, *p* < 0.001), trust (*β* = −3.456, *p* < 0.001), cohesion (*β* = −2.112, *p* < 0.001), reciprocity (*β* = −1.117, *p* < 0.001), and social participation (*β* = 0.788, *p* < 0.01). After adjusting for sociodemographic covariates in model 2, loneliness was still significantly associated with social participation (*β* = 0.658, *p* < 0.05), social support (*β* = −1.081, *p* < 0.001), social connection (*β* = −2.394, *p* < 0.001), trust (*β* = −3.143, *p* < 0.001), and cohesion (*β* = −1.879, *p* < 0.001). After adjusting for both sociodemographic covariates and institutionalization in model 3, social participation (*β* = 0.599, *p* < 0.05), social support (*β* = −0.913, *p* < 0.01), social connection (*β* = −2.376, *p* < 0.001), trust (*β* = −3.054, *p* < 0.001), and cohesion (*β* = −1.765, *p* < 0.001) were survived in the loneliness. As shown in model 3, institutionalization also significantly influenced loneliness. After adjusting for sociodemographic covariates, institutionalization, and social capital in model 4, we found that only the interaction of cohesion and institutionalization had a significant effect on loneliness (*β* = −1.999, *p* < 0.01).

**Table 2:** Regression analysis of the effect of social capital and institutionalization on loneliness among older adults.

Variable	Model 1 *β* (*SE*)	Model 2 *β* (*SE*)	Model 3 *β* (*SE*)	Model 4 *β* (*SE*)
Structural social capital
Social participation (ref: low)
High	0.788 (0.265)^**^	0.658 (0.268)^*^	0.599 (0.268)^*^	1.028 (0.848)
Social support (ref: low)
High	−1.120 (0.298)^***^	−1.081 (0.295)^***^	−0.913 (0.298)^**^	−1.247 (0.975)
Social connection (ref: low)
High	−2.716 (0.487)^***^	−2.394 (0.496)^***^	−2.376 (0.494)^***^	−4.134 (1.451)^**^
Cognitive social capital
Trust (ref: low)
High	−3.456 (0.326)^***^	−3.143 (0.326)^***^	−3.054 (0.326)^***^	−4.613 (1.001)^***^
Cohesion (ref: low)
High	−2.112 (0.345)^***^	−1.879 (0.344)^***^	−1.765 (0.345)^***^	1.076 (1.065)
Reciprocity (ref: low)
High	−1.117 (0.332)^***^	−0.654 (0.338)	−0.651 (0.337)	−1.188 (1.077)
Age (ref: 60–69)
70–79		−0.357 (0.322)	−0.413 (0.321)	−0.410 (0.325)
80-		0.418 (0.394)	0.172 (0.400)	0.178 (0.409)
Sex (ref: male)
Female		−0.108 (0.266)	−0.116 (0.265)	−0.090 (0.266)
Education (ref: primary or below)
Middle		1.197 (0.340)^***^	1.246 (0.339)^***^	1.273 (0.340)^***^
High or above		0.852 (0.387)^*^	0.820 (0.386)^*^	0.787 (0.387)^*^
Marital status (ref: married)
Others		0.784 (0.311)^*^	0.676 (0.312)^*^	0.658 (0.312)^*^
Monthly income (RMB) (ref: 0–2,999)
3,000–4,999		−0.695 (0.343)^*^	−0.795 (0.343)^*^	−0.673 (0.346)
5,000–9,999		−1.796 (0.427)^***^	−1.678 (0.427)^***^	−1.608 (0.428)^***^
10,000-		−1.381 (0.567)^*^	−1.112 (0.571)	−1.002 (0.574)
Number of children (ref: no)
1–2		0.379 (0.980)	0.320 (0.977)	0.129 (0.980)
3-		1.311 (1.031)	1.414 (1.027)	1.182 (1.031)
Having chronic diseases (ref: no)
Yes		0.088 (0.323)	−0.028 (0.324)	0.055 (0.326)
Place of residence (ref: living in community dwellings)
Living in nursing homes			0.992 (0.312)^**^	1.173 (0.567)^*^
Interactions
Social participation*institution				−0.320 (0.528)
Social support*institution				0.208 (0.598)
Social connection*institution				1.423 (1.067)
Trust *institution				1.103 (0.667)
Cohesion*institution				−1.999 (0.697)^**^
Reciprocity*institution				0.358 (0.669)
*R* ^2^	0.318	0.348	0.353	0.359
Adjusted *R*^2^	0.315	0.339	0.344	0.346

^*^p < 0.05, ^**^p < 0.01, ^***^p < 0.001. Ref., reference group.

### The influence of sociodemographic factors and social capital on loneliness among older adults living in community dwellings and nursing homes

Hierarchical regression models were applied to investigate sociodemographic factors and social capital of loneliness in each studied group (Table 3). For sociodemographic factors, age, monthly income, and marital status were associated with loneliness among older adults living in community dwellings, while age and education were associated with loneliness among older adults living in nursing homes. Social capital was strongly associated with loneliness in both groups. As is clear from model 1 (unadjusted), social participation (*β* = 0.778, *p* < 0.05), social support (*β* = −1.079, *p* < 0.05), social connection (*β* = −2.890, *p* < 0.001), trust (*β* = −3.671, *p* < 0.001), and reciprocity (*β* = −0.962, *p* < 0.05) were negatively associated with loneliness in older adults living in community dwellings. For nursing home residents, trust (*β* = −2.766, *p* < 0.001), cohesion (*β* = −3.274, *p* < 0.001), and reciprocity (*β* = −0.904, *p* < 0.05) were negatively associated with loneliness. After controlling for the covariates in model 2, loneliness among older adults living in community dwellings came to be associated with social support (*β* = −0.953, *p* < 0.05), and social connection (*β* = −2.390, *p* < 0.001), trust (*β* = −3.283, *p* < 0.001), cohesion (*β* = −0.941, *p* < 0.05). Among older adults in nursing homes, social support (*β* = −0.805, *p* < 0.05), trust (*β* = −2.212, *p* < 0.001), and cohesion (*β* = −2.982, *p* < 0.001) were associated with loneliness.

**Table 3:** Regression analysis of social capital and loneliness among older adults living in community dwellings and nursing homes.

Variable	**Older adults in community dwellings**		**Older adults in nursing homes**	
	Model 1 *β* (*SE*)	Model 2 *β* (*SE*)	Model 1 *β* (*SE*)	Model 2 *β* (*SE*)
Structural social capital
Social participation (ref: low)
High	0.778 (0.381)^*^	0.671 (0.379)	0.546 (0.367)	0.351 (0.378)
Social support (ref: low)
High	−1.079 (0.443)^*^	−0.953 (0.437)^*^	−0.798 (0.407)	−0.805 (0.403)^*^
Social connection (ref: low)
High	−2.890 (0.563)^***^	−2.390 (0.573)^***^	−1.625 (0.922)	−1.535 (0.943)
Cognitive social capital
Trust (ref: low)
High	−3.671 (0.421)^***^	−3.283 (0.425)^***^	−2.766 (0.513)^***^	−2.212 (0.515)^***^
Cohesion (ref: low)
High	−0.886 (0.460)	−0.941 (0.456)^*^	−3.274 (0.523)^***^	−2.982 (0.528)^***^
Reciprocity (ref: low)
High	−0.962 (0.486)^*^	−0.902 (0.482)	−0.904 (0.457)^*^	−0.465 (0.473)
Age (ref: 60–69)
70–79		−0.761 (0.409)		0.702 (0.534)
80-		−1.248 (0.630)^*^		1.868 (0.591)^**^
Sex (ref: male)
Female		−0.633 (0.382)		0.393 (0.369)
Education (ref: primary or below)
Middle		0.964 (0.502)		1.660 (0.465)^***^
High or above		0.368 (0.505)		0.754 (0.228)
Marital status (ref: married)
Others		1.628 (0.445)^***^		−0.436 (0.455)
Monthly income (RMB) (ref: 0–2,999)
3,000–4,999		−1.663 (0.562)^**^		0.197 (0.445)
5,000–9,999		−2.241 (0.564)^***^		−0.715 (0.718)
10,000-		−1.785 (0.681)^**^		1.237 (1.263)
Having children (ref: no)
1–2		−0.203 (1.395)		0.579 (1.355)
3-		0.199 (1.465)		2.160 (1.431)
Having chronic diseases (ref: no)
Yes		−0.049 (0.398)		0.862 (0.558)
*R* ^2^	0.279	0.323	0.245	0.294
Adjusted *R*^2^	0.272	0.303	0.238	0.274

^*^p < 0.05, ^**^p < 0.01, ^***^p < 0.001. Ref., reference group.

## Discussion

In this study, we observed differences in the associations between social capital and loneliness for the older adults living in community dwellings and nursing homes, and demonstrated that social capital had great impact on older adults’ loneliness in both living settings, indicating the role of social capital in protecting the mental health of older adults. These imply that relevant social capital plays an important role in protecting the mental health of older adults.

Generally, older adults living in nursing homes were more likely to feel lonely than those living in community dwellings, and institutionalization significantly contributed to their level of loneliness, after controlling for sociodemographic and social capital variables. This finding is consistent with previous literature (9, 42, 43). Most older adults are willing to live in the community for aging, which allows them to maintain a sense of autonomy, independence, security, familiarity, and connection (29, 44, 45). Chinese society is a collectivist, and acquaintance-based society (46), in which older adults value social relationships and prefer to live with families, relatives, and friends (29). When older adults move to nursing homes, they are usually at high risk of losing close ties (partners, other relationships, friends, and neighbors) and are more likely to experience loneliness (7).

Another finding of this study was that sociodemographic factors also contributed to loneliness among older adults in two different residential settings. Older adults living in community dwellings without a partner and with low income tended to feel more loneliness, as was observed in previous studies (30, 47). However, a finding that differs from previous studies is that institutionalized older adults with a middle level of education experienced higher degrees of loneliness compared to those with less than primary school or education, while this was not found among those with a high level of education. One possible explanation is that older adults with middle education degrees lack peers with a common language in institutions (7), but older adults with higher educational attainment have more opportunities to extend their social relationships and have more social contacts (48).

Our study examined the differences between institutionalized and noninstitutionalized older adults on six components of social capital. Compared with community-dwelling older adults, institutionalized older adults had lower degrees of social support, social connection, trust, cohesion, and reciprocity. Social capital, often defined as socially supportive resources embedded in social systems, can be considered a protective factor for older adults. Most urban Chinese older adults were willing to live in community for aging as they can obtain high levels of family and community capital there (29). Institutionalized older adults generally have lower social capital. Hence, social capital enhancement measures should be better integrated into health-related policies for them.

Specifically, for various indicators of social capital, our study revealed that social support, trust, and cohesion were negatively related to loneliness in both groups of older adults.

Social support, defined as the frequency with which older adults receive assistance from other people or groups during difficult times (39), represents older adults’ beliefs about the possible help their relationship networks may provide as well as the quantity and quality of this help (49). Social support can help the older adults obtain material and spiritual support, feel love and care, reduce the occurrence of psychological problems, and improve their quality of life (50). Among older adults in the community, the family and kinship system is the most important source of social support. Family members provide food, care and financial assistance to older people. Close interpersonal relationships are very important for the long-term development of a sense of familiarity and security (29). In addition, friends and neighborhood committees are important support resources (51), and they can provide the older adults with necessary financial and psychological support to reduce loneliness. Older adults who move into nursing homes have new social opportunities, and the social support provided by peers and staff within the institution is just as important as that provided by family and friends in community (52). Long-term friendships are very important for the well-being of old adults living in nursing homes, and close friends are irreplaceable (53). Because it is quite difficult to develop new friendships within the institution (10). Older adults may benefit from actively seeking social support (54), such as through the expansion of social networks (55, 56), which protect themselves from occurrence of psychological problems.

Trust refers to the degree of belief in family, friends, and neighbors which acts as lubricants in interpersonal interactions (57), as spouses and close friends can evoke feelings of intimacy, security and peace. Trust can bring people together, increase their attention to others (58), promote information exchange and knowledge dissemination, and help establish a good interpersonal relationship (59) to reduce loneliness. Trust in older adults can come not only from relatives or confidants, but also from people who can connect with their feelings and emotions to provide security (60). For the older adults in institutions, caregivers are daily service providers, who have regular contact and close relationship with them (42). Emotional connection with a trusted caregiver can reduce the loneliness of older adults to some extent (61). For older adults in the community, informal care is the main source of nursing, and most caregivers are their close relatives (7). Having a trusted family member provide care may prevent loneliness. Higher levels of trust in the community may promote information dissemination, service utilization, and healthy behaviors (62, 63), and reduce loneliness among the older adults.

Cohesion is the feeling that an individual derives strength from the group to which they belong (64), and it is an intrinsic link between the individual and the group. With the growth of age and the decline of mobility, older adults prefer to belong to a certain group and be recognized and accepted by the group, which has important implications for their quality of life and happiness (10). Within a well-established group, the older adults can share information and exchange views with each other, participate in collective social activities, play similar roles, and receive love and help in this process, thereby generating and sharing collective interests (29, 59), counteracting social loneliness and adjusting negative psychology (42). Cohesion is also capable of spawning positive mental states, increasing feelings of acceptance (65), security or self-efficacy (66), motivating individuals to improve their behavior (67, 68), and to help reduce loneliness. It is much easier for older adults who live in community to integrate into their community groups and create a sense of acceptance via interacting with neighbors and caring for community affairs (65). In our study, the interaction of cohesion and institutionalization was significantly associated with loneliness. We speculated that older adults are more eager to integrate because they are in an institutional group that is far from their families. If older adults have a sense of belonging and familiarity with the institution (12), the atmosphere within the institution is harmonious, and there is a greater sense of security and trust among the members (69), which reduces the feeling of loneliness.

In addition, social connection influenced loneliness among older adults living in community dwellings other than in nursing homes. Social connection indicates the degree of communication between older adults and their family members, friends, and neighbors. Older adults can improve their social adaptation by establishing contacts and acquiring more information through communication (70), which helps enhance their sense of self-worth. This can, in turn, promote their health and reduce their feelings of loneliness. Conversely, a lack of interpersonal interactions and social activities can lead to psychological problems and a feeling of emotional loss (71, 72). Building connections, especially face-to-face connections, can provide a sense of security to older people (42), regulate their physiological responses, and mitigate their negative emotions (73). The decline of physical and cognitive functions in older adults may cause them to become more dependent on their social contacts (74). Individuals who engage more frequently with members of their social relationship networks can improve the quantity and quality of their interactions, and are more likely to experience less loneliness (75). However, social connection was not found to be associated with loneliness among older adults living in nursing homes. We inferred that most of older adults living in nursing homes investigated may generally have low levels of social connection, because of their limited mobility and reduced contact with others after living in nursing homes (42). Moreover, measures for the prevention and control of the COVID-19 pandemic further limited their social interaction and activities.

In this study, reciprocity and social participation were not significantly related to older adults’ loneliness . Generalized reciprocity refers to an ongoing exchange relationship that is unreciprocated or unbalanced at any given time, but where both parties expect that benefits given now should be reciprocated in the future (76). In particular, reciprocity was not observed in the adjusted regression models, and its role may depend on presence of other dimensions of social capital, as well as the covariates involved. Reciprocity refers to mutual help between individuals. That is, anyone who receives help should return it to the helper or another person (77). Research has pointed out that reciprocity indirectly influences health by helping maintain social networks and social participation. If people believe there is no loss in doing so, they are more likely to contribute to the group and subsequently obtain health benefits (76). Social participation was not found to be associated with loneliness in this study, which is inconsistent with the results of a previous study (19). Social participation is a set of social activities performed voluntarily by an individual to interact with others, and these activities include participation in sports, recreation, cultural programs, and neighborhood associations (78). The non-significant results of social participation in our study may be due to its generally low levels of all participants. As the survey for this study was conducted during the COVID-19 pandemic, epidemic prevention measures restricted older adults from participating in such organized activities (79, 80), and this might have led to a low, or even no, association between reciprocity and loneliness.

In summary, our results indicated that the difference in the sociodemographic characteristics as well as the levels of social capital between older adults living in nursing homes and those in community dwellings could be introduced to explain the difference in the levels of loneliness between them.

## Future implication

According to our research, social capital and institutionalization were the main determinants of loneliness among older adults. Thus, to alleviate loneliness among the older adults in community dwellings and in nursing homes, relevant measures should be taken to improve social capital of the old adults, particularly focusing on social support, trust, and cohesion. In the community, the following actions can be taken: (i) strengthen social support for older adults by providing regular contact, care, and companionship from family and social workers; (ii) promote intergenerational integration by providing opportunities for older adults to participate in recreational activities with different age groups, especially by maintaining close relationships with the younger generation (81), in order to cultivate trust and social connections; (iii) communication social skills training (e.g., telephone or internet use). While, the following solutions are available in nursing homes: (i) provide care services based on the needs of older adults; (ii) enhance emotional communication between older adults and caregivers; (iii) facilitate meaningful social interaction, especially between people with common interests (e.g., set up book clubs, offer trips to senior centers).

## Limitations

This study has a few limitations. First, the data were collected from three cities in Zhejiang Province, and this may limit the generalizability of the findings. Second, this was a cross-sectional study that did not clarify the causal relationship between social capital and loneliness. Longitudinal or controlled randomized trials should be considered in future studies to address this. Third, part of the data in the study was based on self-reports, which might cause bias in the responses. Fourth, in our analysis, we converted social capital scores into dichotomous variables, which may led to a loss of information. Fifth, the present study used convenient sampling, which may introduce selection bias and limit the representativeness of the sample. Future studies with expanded investigative sites and large sample sizes are needed.

## Conclusion

This study indicated that components of social capital, particularly social support, trust, and cohesion, were significantly associated with loneliness among institutionalized and noninstitutionalized older adults. In addition, institutionalization itself and the interaction of cohesion and institutionalization also had an impact on loneliness among older adults. Health-related policies should help older adults gain more social capital to reduce their loneliness. This is particularly critical for older adults living in nursing homes, who have higher levels of loneliness and lower social capital than noninstitutionalized older adults.

## Data availability statement

The raw data supporting the conclusions of this article will be made available by the authors, without undue reservation.

## Ethics statement

The studies involving human participants were reviewed and approved by Ethics Committee of Hangzhou Normal University (No. 20210002). The patients/participants provided their written informed consent to participate in this study.

## Author contributions

YC and DW contributed to conception and design of the study. ML organized the database. YCZ, YH, and HC performed the statistical analysis. YCZ, YC, and SZ wrote the first draft of the manuscript. YYZ, SY, and XW wrote sections of the manuscript. All authors contributed to manuscript revision, read, and approved the submitted version.

## Funding

This research was funded by Zhejiang Provincial Department of Education (Grant Number Y202250035), and the National Natural Science Foundation of China (Grant Number 71904037).

## Conflict of interest

The authors declare that the research was conducted in the absence of any commercial or financial relationships that could be construed as a potential conflict of interest.

## Publisher’s note

All claims expressed in this article are solely those of the authors and do not necessarily represent those of their affiliated organizations, or those of the publisher, the editors and the reviewers. Any product that may be evaluated in this article, or claim that may be made by its manufacturer, is not guaranteed or endorsed by the publisher.
