# Supplementary material for: Social capital and loneliness among older adults in community dwellings and nursing homes in Zhejiang Province of China
Source: Front Public Health. 2023 May 19;11:1150310. doi: 10.3389/fpubh.2023.1150310 (PMC10237354; doi:10.3389/fpubh.2023.1150310)
Supplement: Supplementary file 1 [file Data_Sheet_3.PDF]

Supplementary Material

Social Capital and Loneliness among Older Adults in Community Dwellings and Nursing Homes in Zhejiang Province of China

Yan Chen†*, Yuchen Zhou†, Min Li, Yanyan Hong, Hongkun Chen, Shanshan Zhu, Yiying Zhou, Shuangyu Yang, Xianlan Wu, Dahui Wang

†These authors contributed equally to this work and share first authorship

*** Correspondence:** Yan Chen: 20130046@hznu.edu.cn

Supplementary Tables

Table 1 Assessment of Loneliness.

**Loneliness**	I lack companionship.	①Never ②seldom ③usually ④always
	There is no one I can turn to.	①Never ②seldom ③usually ④always
	I am an outgoing person.	①Never ②seldom ③usually ④always
	I feel left out.	①Never ②seldom ③usually ④always
	I feel isolated from others.	①Never ②seldom ③usually ④always
	I can find companionship when I want it.	①Never ②seldom ③usually ④always
	I am unhappy being so withdrawn.	①Never ②seldom ③usually ④always
	People are around me but not with me.	①Never ②seldom ③usually ④always

Table 2 Measurement of Social Capital.

**Social Participation**	In the past 12 months, how often would you participate in formal groups (party or democratic parties' elections, etc.)?	①Never ②Seldom ③Usually ④Often ⑤More Often
	In the past 12 months, how often would you participate in informal groups (square dance, interest clubs, etc.)?	①Never ②Seldom ③Usually ④Often ⑤More Often
	In the past 12 months, how often would you be community/institutional volunteer (coordinator, corridor manager, etc.)?	①Never ②Seldom ③Usually ④Often ⑤More Often
	In the past 12 months, how often would you take part in community/institutional services (health lecture, cultural activities etc.)?	①Never ②Seldom ③Usually ④Often ⑤More Often
**Social Support**	When you are in trouble, is there someone that provides you with mental support (i.e., comfort you)?	①Never ②Seldom ③Usually ④Often ⑤More Often
	When you are in trouble, is there someone that provides you with material support (i.e., lend you money)?	①Never ②Seldom ③Usually ④Often ⑤More Often
	When you are in trouble, are there any formal or informal groups that provide you with mental support (i.e., comfort you)?	①Never ②Seldom ③Usually ④Often ⑤More Often
	When you are in trouble, are there any formal or informal groups that provide you with material support (i.e., lend you money)?	①Never ②Seldom ③Usually ④Often ⑤More Often
**Social Connection**	How often do you contact with your children?	①Never ②Seldom ③Usually ④Often ⑤More Often
	How often do you contact with your relatives?	①Never ②Seldom ③Usually ④Often ⑤More Often
	How often do you contact with your friends/ neighbors?	①Never ②Seldom ③Usually ④Often ⑤More Often

**Trust**	Do you trust in your family members?	①Never ②Seldom ③Usually ④Often ⑤More Often
	Do you trust in your friends?	①Never ②Seldom ③Usually ④Often ⑤More Often
	Do you trust in someone who lives within one community/nursing home?	①Never ②Seldom ③Usually ④Often ⑤More Often
**Cohesion**	Do you care about what happened in your community/nursing home?	①Never ②Seldom ③Usually ④Often ⑤More Often
	Do you think the community/nursing home is more harmonious?	①Never ②Seldom ③Usually ④Often ⑤More Often
	Do you like the community/nursing home you live now?	①Never ②Seldom ③Usually ④Often ⑤More Often
	Do you have a feeling of being in the community/nursing home?	①Never ②Seldom ③Usually ④Often ⑤More Often
	Do you feel reluctant, if you have to move away from the community/nursing home lived now?	①Never ②Seldom ③Usually ④Often ⑤More Often
**Reciprocity**	When your relatives are in trouble, will you provide help to them?	①Never ②Seldom ③Usually ④Often ⑤More Often
	When your friends/ neighbors are in trouble, will you provide help to them?	①Never ②Seldom ③Usually ④Often ⑤More Often
	When some strangers are in trouble, will you provide help to them?	①Never ②Seldom ③Usually ④Often ⑤More Often
